# Laboratory-based assessment of quantitative PCR to describe Arctic fish assemblages in surface water affected by acid rock drainage

**DOI:** 10.1371/journal.pone.0362179

**Published:** 2026-11-10

**Authors:** Laura C. Scott, Andrew M. Ramey, Ann E. Riddle-Berntsen, Brett A. Poulin, Joshua C. Koch, Jonathan A. O’Donnell, Michael P. Carey

**Affiliations:** 1 U.S. Geological Survey, Alaska Science Center, Anchorage, Alaska, United States of America; 2 Department of Environmental Toxicology, University of California, Davis, California, United States of America; 3 National Park Service, Arctic Network, Anchorage, Alaska, United States of America; UTRGV: The University of Texas Rio Grande Valley, UNITED STATES OF AMERICA

## Abstract

Permafrost thaw has been identified as a cause of metal mobilization from acid rock drainage and transport to Arctic rivers. Elevated metal concentrations and precipitation of Fe(III) minerals (i.e., rusting) in surface waters deteriorates water quality and raises questions about ecosystem effects, especially to the health of fish species. Environmental DNA (eDNA) analyses could be used for assessing impacts of acid rock drainage to fish assemblages but may be prone to biases like polymerase chain reaction (PCR) inhibition. This study tested whether water from Arctic rivers affected by acid rock drainage influenced the efficacy of quantitative PCR (qPCR) for detecting fish. Water samples from rivers influenced by acid rock drainage were collected, characterized, and sterilized. Fish tissue was then added and mixed, and aliquots were analyzed using qPCR to assess species-specific detection sensitivity, specificity, and inhibition. In experiments, qPCR exhibited perfect species-specific assay sensitivity. There was little evidence for qPCR inhibition, whereas amplification efficiency was higher in approximately half of the samples. In mixed species assemblages, the proportion of species-specific target genes detected was significantly correlated to the proportion of tissue present for most targets. Results indicate that qPCR-based approaches, such as those often used in eDNA analyses, may help describe Arctic fish species assemblages within surface waters affected by acid rock drainage. Evaluation of qPCR methods described in this laboratory-based study using field-collected samples could help determine the effectiveness of these approaches in natural systems.

## Introduction

Rapid warming of the Arctic is creating disturbances from permafrost thaw in terrestrial and aquatic ecosystems. An emergent phenomenon is the mobilization of metals from thawing permafrost to streams and rivers, degrading their water quality with potential consequences for aquatic food webs [1]. The resulting orange discoloration, often termed “rusting rivers,” is caused by particulates in surface water and on stained stream beds, namely of Fe(III) minerals [2]. Previous investigations have described the spatial extent and potential toxicity of this process within Arctic Alaska [1,3–5]; however, there are many uncertainties about the effect of acid rock drainage on aquatic ecosystems. In particular, key questions remain about the physical and chemical effects of short- and long-term elevated metal concentrations and lower pH on aquatic communities. The metals mobilized to streams and rivers across Alaska’s Brook Range, such as cadmium (Cd), copper (Cu), and nickel (Ni), can be acutely toxic and have a multitude of other negative influences on aquatic life, including effects on stream sediment and water clarity [1,4,6,7].

A primary concern regarding impacts of rusting rivers is the potential effects of metals and minerals on fish abundance, distribution, and health, especially species important for commercial, recreational, and subsistence harvest within the Arctic [5]. These species include Dolly Varden (*Salvelinus malma*), Chum Salmon (*Oncorhynchus keta*), and Arctic Grayling (*Thymallus arcticus*). Dolly Varden, Chum Salmon, and Arctic Grayling inhabit rivers with degraded water quality caused by rusting rivers [4].

Arctic Grayling are freshwater residents that occur widely across the region and are important recreational and subsistence species. Dolly Varden is an important recreational and subsistence species which may be impacted by acid rock drainage because this species relies on freshwater streams and rivers for reproductive, emergence, and rearing stages of its life history [8]. Dolly Varden inhabiting Arctic Alaska spawn in freshwater streams where young fish typically rear for two to five years before migrating out to productive coastal areas to feed, allowing them to grow rapidly. There are also freshwater resident Dolly Varden. Chum Salmon is an important commercial and subsistence species that also spawns and emerges in freshwater, including watersheds potentially impacted by acid rock drainage [4]. In contrast to Dolly Varden, young Chum Salmon migrate to ocean habitats soon after emergence where they grow for three to five years; there is no resident phenotype of the species in Arctic Alaska. Though not a commonly harvested fish species, Slimy Sculpin (*Cottus cognatus*) is another ecologically important freshwater resident taxon widely distributed throughout Arctic Alaska. Slimy Sculpin typically exhibit a small home range. Given their low trophic position in freshwater food webs and contrasting life history relative to the other previously mentioned Arctic fish species, they could be differentially responsive to acid rock drainage [9].

A major challenge to assessing potential effects of acid rock drainage on fish abundance, distribution, and health within Arctic Alaska is the large spatial scale at which this phenomenon is occurring [1]. Therefore, cost- and time-efficient tools are needed to facilitate assessment and quantification of changes across vast and dynamic Arctic landscapes. While traditional sampling methods for fish provide information on biological and physiological consequences of water quality changes from permafrost characteristics [10], non-invasive environmental deoxyribonucleic acid (eDNA) techniques may provide more cost- and time-effective approaches to sample across a spatially extensive region of Arctic Alaska [11,12]. Collecting eDNA samples generally requires less time at a field location compared to traditional fish capture methods, relatively small water volumes (i.e., 0.5–5 liters can be filtered quickly), and samples can be readily transported for analysis. Moreover, eDNA has the potential to be a less-biased sampling method than selective fishing gear in heavily impaired streams, such as those affected by acid rock drainage, where water quality could affect encounter probability for rare organisms or those unlikely to be captured using traditional methods [13,14]. When using eDNA techniques to assess the composition of fish communities, sampling techniques can be consistently applied to different sized streams and rivers to assess impacts on watersheds at various spatial scales.

Quantitative PCR (qPCR) is a commonly used molecular approach for the analysis of eDNA samples. Here we report on laboratory-controlled experimental efforts to test the utility of qPCR to detect DNA from four fish species common within Arctic Alaska and found in rivers affected by acid rock drainage as mapped in O’Donnell et al. (2024) [1] and Carey et al. (2026) [5]). Quantitative PCR is susceptible to inhibition from compounds present in environmental samples, including metals and minerals, potentially biasing results and reducing reproducibility [15–18]. For this reason, we sought to determine the extent to which these potential effects may bias qPCR analyses to describe fish assemblages in surface waters impacted by acid rock drainage and associated Fe(III) minerals. Therefore, we systematically assessed the amplification and detection efficiency of previously developed qPCR primers for Arctic Grayling, Chum Salmon, Dolly Varden, and Slimy Sculpin in impacted water samples. This study lays the groundwork for potential future application of eDNA analysis in field settings to assess the impacts of rusting rivers on fishes with differing life histories and freshwater dependency in Arctic Alaska.

## Materials and methods

### Study design overview

To evaluate the applicability of qPCR for detecting four fish species common to Arctic Alaska where acid rock drainage has been observed [1], we designed a series of experiments where river water samples collected from field sites affected by natural acid rock drainage [1,7] were inoculated with known quantities of fish tissue, mixed, and filtered (details provided in subsequent text). DNA was extracted from filters, diluted in series, and tested using qPCR targeting mitochondrial gene targets for Arctic Grayling, Chum Salmon, Dolly Varden, and Slimy Sculpin. Two sets of tests were conducted to: (1) assess species-specific detection and amplification efficiency in isolation and (2) assess species-specific detection and amplification efficiency in simulated mixed species assemblages. Analyses were conducted to provide detailed information on assay specificity, sensitivity, and amplification efficiency.

### Collection of surface water from Arctic rivers affected by acid rock drainage

Water samples were collected for experimentation and characterization from rivers in the western Brooks Range of Alaska in Noatak National Preserve and Gates of the Arctic National Park and Preserve (S1 Table). Natural acid rock drainage driven by permafrost thaw is the primary mechanism of the degraded water quality on National Park lands in remote Alaska where the water samples were collected [1,7]. Both the Kugururok River and Igning River have seeps with reported conditions of low pH and elevated metal concentration that enter neighboring mainstem rivers [1]. Unfiltered water was collected in 2l Nalgene glycol-modified polyethylene terephthalate (PETG) bottles with no head spaces during the open-water period in August 2024. Samples were collected under National Park Service Permit (WEAR-2023-SCI-0004). Discrete *in situ* measurements of stream temperature, pH, specific conductivity, and dissolved oxygen were collected using a YSI ProDSS multiparameter meter (YSI Incorporated, Yellow Springs, OH, USA). We calibrated the pH sensor (accuracy ±0.2 units) in the laboratory prior to in situ measurements using a 3-point calibration (pH at 4, 7, and 10). Specific conductivity was calibrated using a one-point method (1,000 µS cm-1). Dissolved oxygen was calibrated at one point using the 100% saturation method. For chemical characterization, unfiltered subsamples were collected for total elemental quantification (collected in PETG bottles, preserved in the field to 1% v v^-1^ Omnitrace Corp (Palm Beach Gardens, FL, USA) nitric acid (HNO_3_)) and dissolved inorganic carbon (DIC) (collected in 60-mL syringes and stored at 4 °C) and filtered samples (0.45-μm high-capacity capsule filter, Geotech (Denver, CO, USA), acrylic copolymer) for major cations (1% v v^-1^ Omnitrace HNO_3_ stored at room temperature), trace element (1% v v^-1^ Omnitrace HNO_3_ stored at room temperature), and major inorganic anion quantification (collected in brown high density polyethylene (HDPE) bottles and stored at 4 °C).

Total major and trace element concentrations on unfiltered water samples were measured after digestion (3% HNO_3_, 8-hour digest at 65 °C) and matrix adjustment (to 2% HNO_3_, 0.5% hydrochloric acid (HCl)) by Inductively Coupled Plasma-Mass Spectroscopy (ICP-MS) using U.S. EPA method 3015A (2007) and an iCAP RQ ICP-MS (Thermo Fisher Scientific, Waltham, MA, USA) for: ^9^Be, ^27^Al, ^51^V, ^52^Cr, ^55^Mn, ^57^Fe, ^59^Co, ^60^Ni, ^63^Cu, ^66^Zn, ^75^As, ^77^Se, ^89^Y, ^107^Ag, ^111^Cd, ^137^Ba, ^139^La, ^140^Ce, ^141^Pr, ^146^Nd, ^163^Dy, ^205^Tl, ^206^Pb, ^207^Pb, ^208^Pb, ^232^Th, and ^238^U. Filtered water samples were matrix-adjusted and analyzed using ICP-MS. Major cations (calcium (Ca^2+^), magnesium (Mg^2+^), sodium (Na^+^) and potassium (K^+^)) were measured on filter-acidified metal samples at the University of California Davis Analytical Laboratory following the U.S. Environmental Protection Agency (EPA) Method 200.7 (1994) [19]. DIC measurements were made on a TOC-VCSH Combustion Analyzer (Shimadzu Corp., Kyoto, Japan). Major inorganic anions (chloride (Cl^-^), sulfate (SO_4_^2-^), nitrate (NO_3_^-^) analyzed by ion chromatography (Metrohm A Supp 7 column).

### Geochemical modeling of mineral precipitation

The Geochemist’s Workbench (Community Edition 18.0; https://community.gwb.com/community_download.php) was used to model the saturation indices of minerals to identify minerals that were supersaturated (i.e., predicted to precipitate) across the river waters. The modeling supported interpretation of the particulate mineral phases likely present in the river samples. Saturation indices were calculated using the SpecE8 module with the default thermodynamic database (thermos.tdat). Site-specific chemistry input included pH, water temperature (°C), and concentrations of DIC (as mgC L^-1^), Ca^2+^, Mg^2+^, Na^+^, K^+^, SO_4_^2-^, Cl^-^, and select metals (Al (as Al^3+^), Cu (as Cu^2+^), Zn (as Zn^2+^), Fe (as Fe^3+^), Ni (as Ni^2+^), Pb (as Pb^2+^), Mn (as Mn^2+^), Co (as Co^2+^), and Ba (as Ba^2+^)). The saturation indices (log Q K^-1^) of minerals were used to interpret the particulate mineral composition of modeled waters. The charge imbalance of simulated waters was ≤ 6.4%.

### Quantitative PCR optimization

Quantitative PCR primers specific to Arctic Grayling [20], Chum Salmon [21], Dolly Varden [20], and Slimy Sculpin [20] were tested as both probe-based and SYBR green assays (S2 Table). Because SYBR green assays can have non-specific amplification, high resolution melt analysis was conducted in these assays [21]. Thermal cycling conditions and reaction mixtures in 15-µL volumes for the probe-based assays were used according to Rodgers et al. (2018) [20] for Arctic Grayling, Dolly Varden, and Slimy Sculpin and Homel et al. (2020) [20] for Chum Salmon: DNA denaturation for 10 minutes at 95°C followed by 45 cycles of 95°C denaturation for 15 seconds then a one minute annealing and extension at 60°C. The thermal cycling conditions used in SYBR assays were as follows: Uracil DNA Glycosylase activation at 50°C for 2 minutes then 95°C for 10 minutes, 40 amplification cycles of 95°C for 15 seconds followed by 60°C for 1 minute, and melt curve analysis starting at 95°C for 15 seconds, 60°C for 1 minute and increasing to 95°C at 0.1 °C per second. SYBR assays were done in 15-µL volumes with the same primer concentrations as probe assays, 10 µL of 2x PowerUp SYBR Master Mix (Applied Biosystems, Waltham, MA, USA), and nanopure water. All qPCR assays were run on a QuantStudio 3 machine (Applied Biosystems). Synthetic DNA templates, gBlocks (Integrated DNA Technologies, Inc., Coralville, IA, USA), were used to generate standard curves and test probe-based and SYBR assays. Templates were reconstituted per manufacturer instructions and diluted ten-fold from 10^7^ gene copies/µL to 0.1 copies/µL.

Standard curves for each target were generated after three independent qPCR runs of triplicates for each gene copy concentration. The limit of detection for each assay was determined by the gene copy concentration which had amplification above the threshold and the correct melt temperature in all replicates during standard curve generation. Amplification efficiency was calculated for each assay using Equation 1.


$$\begin{array}{c} \;Efficiency=\left({\text{1}\text{0}}^{\left(-\frac{\text{1}}{slope}\right)}\right)-\text{1} \end{array}$$ (1)

After generating preliminary standard curves with synthetic templates, only SYBR assays were used because they demonstrated greater amplification efficiency (Table 1).

**Table 1:** Standard curves for SYBR and probe-based qPCR assays for four fish species common throughout Arctic Alaska.

Fish Species	SYBR				Probe			
	Standard Curve Equation	R^2^	Efficiency	Limit of Detection	Standard Curve Equation	R^2^	Efficiency	Limit of Detection
Arctic Grayling (*Thymallus arcticus*)	y = -3.5314x + 30.974	0.9992	0.9194	0.1 copy/µl	y = -3.6855x + 40.866	0.9977	0.8677	1 copy/µL
Chum Salmon (*Oncorhynchus keta*)	y = -3.3917x + 31.526	0.9984	0.9717	0.1 copy/µl	y = -3.5431x + 35.546	0.9981	0.9153	1 copy/µL
Dolly Varden *(Salvelinus malma*)	y = -3.4602x + 31.791	0.9990	0.9454	0.1 copy/µl	y = -3.6125x + 37.848	0.9999	0.8915	1 copy/µL
Slimy Sculpin (*Cottus cognatus*)	y = -3.4508x + 31.940	0.9995	0.9489	0.1 copy/µl	y = -3.4599x + 35.403	0.9997	0.9455	1 copy/µL

SYBR-based qPCR assays were then tested with DNA extracted from target fish muscle tissue. Fish samples were collected under a National Park Service Permit (WEAR-2023-SCI-0004) and Alaska Department of Fish and Game Aquatic Resource Permit (SF2024−101). The fish collection protocol was approved by the U.S. Geological Survey, Alaska Science Center Institutional Animal Care & Use Committee (2024−02). DNA was extracted from < 25 mg muscle tissue per the manufacturer protocol for the Blood and Tissue Kit (Qiagen) on a Qiacube system (Qiagen, Hilden, Germany). DNA extract concentrations were measured using Quant-iT dsDNA Assay Kit High Sensitivity (Invitrogen, Waltham, MA, USA) on a GloMax-Multi+ Detection System (Promega, Madison, WI, USA) and diluted seven times in a ten-fold series. Starting tissue DNA concentrations ranged from 50 ng/µL to 500 ng/µL. The seven-fold dilution series was designed to capture the quantitative range of the qPCR assays. Following extraction and dilution, qPCR was conducted as described above.

### Assessing qPCR efficacy from river water samples

To assess effects of acid rock drainage on qPCR efficacy, fish tissue (0.01–0.98 mg) was added to sterilized field-collected water samples as well as a nanopure purified water control for subsequent DNA extraction and qPCR testing [21]. Water samples (approximately 2 L each) were taken from five river sites in Arctic Alaska as described previously (S1 Table). The samples spanned a range of pH, anions, and dissolved and particulate metal concentrations, thus representing varying degrees of acid rock drainage input. Differences in river samples were visually apparent by orange discoloration (Fig 1). Prior to experiments, water samples and the control were autoclaved for 120 minutes at 121°C and 15 psi. Change in pH or specific conductance were not observed after water was autoclaved, though alterations in dissolved, particulate, and organic components were not assessed. Metal speciation would not be expected to be adversely affected by autoclaving, because the solubility of minerals will be identical before and after the temperature cycle; therefore, chemical equilibrium would reset the dissolved versus particulate metal distribution. For each of four fish species, muscle tissue was added to aliquots of each water sample at 1:10, 1:100, and 1:1000 fish tissue:water volume ratios in triplicate. Tissue weights and water volumes are included in the associated data release [22]. While these ratios are likely higher than what might be expected in a river, the aim was to produce qPCR results with consistently positive results at each ratio to assess inhibition. Aliquots of river waters with no fish tissue were also included for testing in triplicate to serve as controls. Samples were mixed on a plate shaker at 1200 revolutions per minute for 30 minutes. Muscle tissue weights and water volumes for every replicate are included in Scott et al. (2025) [22]. Water was filtered across an 0.2µm mixed cellulose ester filter (Millipore Sigma, Burlington, MA, USA). DNA was extracted from filters per manufacturer protocols of the DNEasy PowerWater kit (Qiagen, Hilden, Germany) on a Qiacube system (Qiagen, Hilden, Germany). Extracts were diluted four times in a ten-fold series to assess qPCR inhibition. Each extracted sample was run in triplicate using the qPCR SYBR assay as previously described. All qPCR runs included non-template controls and synthetic DNA template positive controls. A sample was considered to have a positive detection of a given fish species if at least two of three qPCR replicates amplified with the correct melt temperature above the standard curve limit of detection. Gene copy concentrations from the starting water sample were estimated using the standard curves.

**Fig 1:**
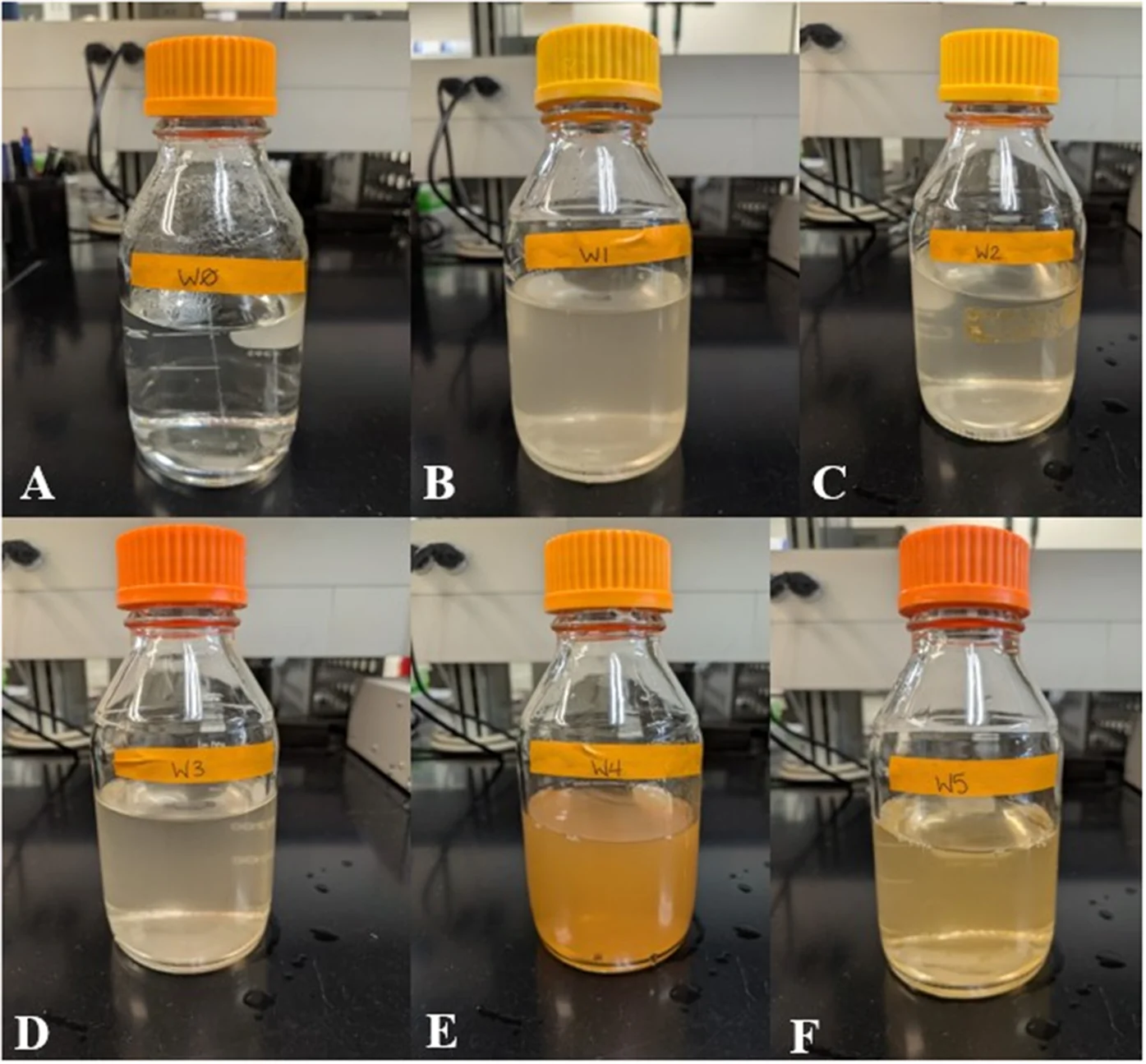
Photographs of unfiltered surface water samples used in this study (Photograph credit: L.C. Scott). W0 (panel A) is the purified water control whereas W1-W5 (panels B-F) depict river water affected by acid rock drainage collected from Arctic Alaska (S1 Table).

To further assess PCR inhibition, synthetic DNA template for bacterial *Vibrio spp.* was added to the DNA extracts from water samples in which no fish tissue had been added. These extracts were diluted four times in a ten-fold series. *Vibrio* spp. reaction mixes were comprised of 10 µL 2x PowerUp SYBR Master Mix (Applied Biosystems), 1 µL of 10 µM forward primer, 1 µL of 10 µM reverse primer, 1 µL of nanopure water, and 2 µL of extracted DNA (S1 Table). A *Vibrio* template was chosen as a PCR inhibition target for this study because *Vibrio* species would not be anticipated to occur within freshwaters of Arctic Alaska [23]. Cycle threshold values (Ct values) were evaluated against the magnitude of the extraction dilution to imitate the previously generated standard curves.

The qPCR amplification efficiency for the diluted DNA extractions was compared to that of the standard curve to assess for qPCR inhibition using the samples from the previously described experiment. For this study, PCR inhibition was defined as a deviation of the experimental amplification efficiency from the standard curve by more than 0.2 (20%), further from 1.00, or an amplification efficiency measuring less than 0.90 or greater than 1.10. While PCR inhibition commonly manifests as lowered amplification efficiency, increased efficiency from standards, particularly greater than 100%, can be evidence of PCR inhibition [24]. The difference between the experimental amplification efficiency of gene targets among water samples was statistically compared to amplification efficiencies from standard curves with one-way analysis of variance (ANOVA) tests conducted in Prism (Graphpad Prism, Boston, MA, USA) using two separate comparisons using a significance level of 0.05. First, amplification efficiency differences were pooled as analytical replicates by water sample and tissue dilution to compare efficiency differences between gene targets, including *Vibrio spp.* The measurements from same water sample and tissue preparation were considered independent observation (Table 2). Secondly, amplification efficiency differences were pooled as analytical replicates by gene target and water dilution to compare differences in efficiency by water sample. Changes in amplification efficiency were compared between gene targets with a one-way ANOVA to determine if the qPCR assays were differentially affected across the water samples.

**Table 2:** Amplification efficiencies of qPCR assays (unshaded cells) using DNA extracts from aliquots of a control (W0) and five riverine water samples (W1-W5) affected by acid rock drainage for five quantitative PCR targets, each run-in triplicate. The difference between the experimental efficiency of the DNA extracts and the efficiency of the standard curve for that qPCR assay is listed below experimental amplification efficiency values in orange shaded cells. Observations with an asterisk denote a PCR inhibition qualifying event.

Target	Water:Fish	Standards	W0	W1	W2	W3	W4	W5
*Vibrio spp.*	NA	0.9146	0.8667	0.9091	0.9831	0.9196	1.0067	0.9868
			0.0479	0.0056	−0.0685	−0.0049	−0.0920	−0.0721
Arctic Grayling (*Thymallus arcticus*)	10	0.9194	0.9447	0.9005	0.7788	0.9008	0.9189	0.8488
			−0.0253	0.0190	0.1406	0.0186	0.0006	0.0707
Arctic Grayling (*Thymallus arcticus*)	100	0.9194	0.9905	0.7775	0.9164	0.8546	0.9373	0.9053
			−0.0710	0.1419	0.0030	0.0649	−0.0178	0.0142
Arctic Grayling (*Thymallus arcticus*)	1000	0.9194	0.9325	0.8642	0.9196	0.8072	0.8552	0.9637
			−0.0131	0.0552	−0.0001	0.1122	0.0643	−0.0443
Chum Salmon (*Oncorhynchus keta*)	10	0.9717	1.0572	1.0503	1.0187	0.9950	0.9376	0.9696
			−0.0855	−0.0786	−0.0470	−0.0233	0.0340	0.0021
Chum Salmon (*Oncorhynchus keta*)	100	0.9717	1.0844	1.0309	1.0619	1.0067	0.9680	0.9228
			−0.1127	−0.0592	−0.0902	−0.0350	0.0037	0.0489
Chum Salmon (*Oncorhynchus keta*)	1000	0.9717	1.0820	1.0498	0.9362	0.9771	0.9417	0.9743
			−0.1103	−0.0782	0.0355	−0.0054	0.0300	−0.0027
Dolly Varden *(Salvelinus malma*)	10	0.9454	0.9499	0.9979	0.9196	0.8724	0.9146	0.9572
			−0.0046	−0.0525	0.0258	0.0729	0.0307	−0.0118
Dolly Varden *(Salvelinus malma*)	100	0.9454	1.0071	1.0269	1.0234	0.9336	1.0591	1.0118
			−0.0618	−0.0816	−0.0781	0.0118	−0.1137	−0.0664
Dolly Varden *(Salvelinus malma*)	1000	0.9454	0.9466	0.9199	0.9073	0.9477	0.7868	0.8917
			−0.0012	0.0254	0.0380	−0.0023	0.1585	0.0537
Slimy Sculpin (*Cottus cognatus*)	10	0.9489	1.0349	1.0354	1.0012	1.1667	1.0008	1.0208
			−0.0860	−0.0865	−0.0523	−0.2178	−0.0519	−0.0719
Slimy Sculpin (*Cottus cognatus*)	100	0.9489	1.0762	1.1511*	1.2263*	1.0000	1.0888	0.9175
			−0.1273	−0.2022	−0.2774	−0.0511	−0.1399	0.0314
Slimy Sculpin (*Cottus cognatus*)	1000	0.9489	1.1048*	1.0080	0.9336	0.9413	0.9665	0.9436
			−0.1559	−0.0591	0.0153	0.0076	−0.0176	0.0053

### Quantitative PCR assay efficacy with simulated mixed species assemblages

The ability of the qPCR assay to accurately detect DNA from multiple fish species present in water affected by acid rock drainage was also tested. Fish tissues, including four target and two off target species (Round Whitefish *Prosopium cylindraceum* and European Pilchard *Sardina pilchardus*), were added to aliquots of water samples, including the purified water control. The off-target species were added to determine if non-specific tissues would impact precision of detection of target species. These species were chosen because of their similar appearance and presence in the region (Round Whitefish) and their consumption during fieldwork (European pilchard), presenting a risk for contamination. Concentrations and compositions of fish muscle tissue (0.21–0.56 mg) mixtures were unique and not repeated across water sample aliquots since initial experiments (described above) indicated water chemistry did not affect qPCR efficacy or efficiency [22]. Three mixtures were generated for each of six water samples (n = 18). Tissues were added to water sample aliquots, mixed, filtered, and DNA extracted as described previously. A second team member who was blind to the previous steps carried out the qPCR assays and analysis. For each mixture and gene target (18 sample aliquots * four fish species; n = 72), the team member assessed whether the qPCR assay correctly detected or did not correctly detect the species-specific gene target. Gene copy concentrations in the original water sample were calculated using the standard curve. For each mixture sample, the proportion of gene copies detected for each fish species was calculated alongside the proportion of fish tissue added by weight for each species. These proportions were compared to determine proportional quantitative accuracy of the qPCR assay in impacted samples compared to a control. Tissue and gene copy proportions were statistically compared using Spearman’s R test and graphed using simple linear regression to produce a trend line using Prism 10 software (Graphpad Prism).

## Results

### Quantitative PCR optimization

DNA extracted directly from tissues of the four target fish species were assessed with the qPCR assay in seven ten-fold dilutions. DNA amplification was successful for all four targets across at least six orders of magnitude dilutions from the original extract (Fig 2). The amplification efficiency for qPCR from tissues was similar to synthetic DNA templates for all four targets. Average melt temperatures from synthetic DNA template and fish tissues were consistently within 1°C of each other (S1 Fig).

**Fig 2:**
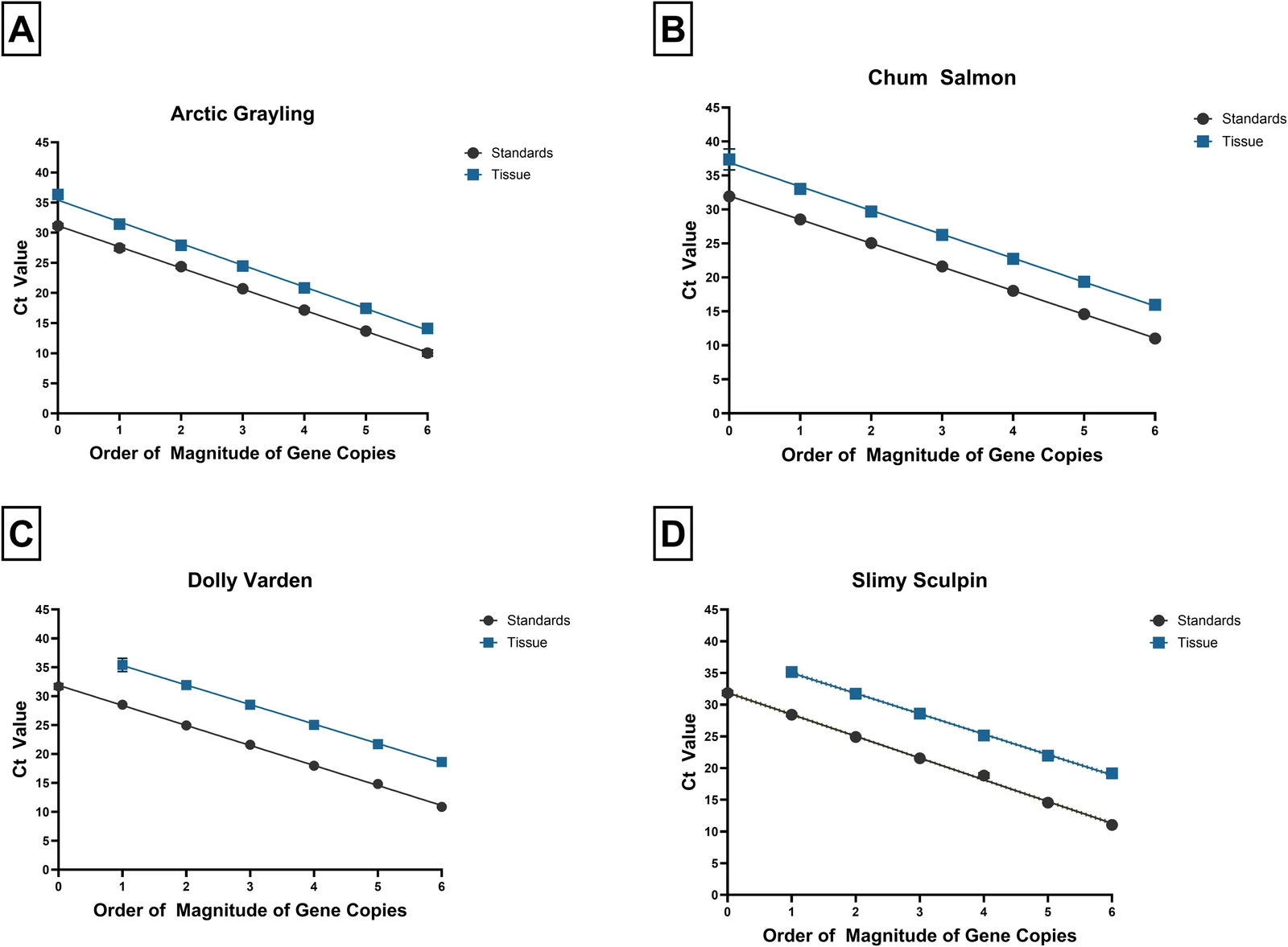
SYBR-based qPCR standard curves for A) Arctic Grayling (*Thymallus arcticus*), B) Chum Salmon (*Oncorhynchus keta*), C) Dolly Varden (*Salvelinus malma*), and D) Slimy Sculpin (*Cottus cognatus*) using synthetic DNA template (black circles) and DNA extracted from fish tissues (blue squares).

### Assessing qPCR efficacy from water samples affected by acid rock drainage

Aliquots of five riverine water samples were mixed with known weights of fish muscle tissues to determine if the inorganic water chemistry and mineral composition (S1 Table) affected qPCR assay sensitivity, specificity, or efficiency. The five river waters varied in pH (4.78–8.03), dissolved ion concentration (specific conductance [SpC] ranged from 1219–3469 μS/cm), and filtered and particulate metal concentrations (S1 Table). Specifically, unfiltered concentrations of major metals (Fe, Al, and Mn) were as high as 87 mg/L (Fe, W4), varied between 12- to 54-fold, and were greater in river samples of lower pH. Mean unfiltered concentrations of trace elements (Ni, Cu, Zn, Pb, Co) exhibited the following hierarchy of concentration: Zn > Ni > Cu > Co > Pb. Under field conditions, geochemical simulations using filtered water data showed that all five waters were supersaturated with a variety of Fe(III) minerals, including Fe_2_O_3_, FeO(OH), and Fe(OH)_3_ (S1 Table). Therefore, the particulates observed in the waters (Fig 1) are likely a mixture of Fe(III) minerals (and potentially other minerals containing Mn or Al) that precipitate under field conditions, similar to observations in other Arctic and boreal rivers affected by acid rock drainage [2]. However,we recognize Fe(III) mineral precipitation may have also occurred during sample transport and storage prior to experimentation. Taken together, the composition of the waters tested spans a range of compositions characteristic of rivers and streams across the Brooks Range affected by varying degrees of acid rock drainage [1,4].

Amplification of DNA targets for four fish species or a *Vibrio* spp. positive control was detected in every sample, dilution, and replicate (S2−S5 Figs) and amplicon melt temperatures were always consistent with positive controls for that target. Three instances of PCR inhibition were detected, all in samples containing Slimy Sculpin tissue, when the amplification efficiency increased above 1.10 (Table 2). Change in amplification efficiency ranged from −0.2774 to 0.1585, averaging −0.0233 with a standard deviation of 0.0769 (Table 2). In 34 of 65 (53.31%) comparisons, amplification efficiencies of DNA targets for fish species or a *Vibrio* spp. positive control were closer to 1.00 when testing extracts from river water samples compared to the standard curve (Table 2).

The differences between amplification efficiencies of standard curves and extracted DNA from sample aliquots using the five riverine waters were not significantly different from one another (one-way ANOVA; df = 5; p = 0.3297; Fig 3). The greatest median difference in amplification efficiency between the standard curve and diluted extracts was produced with samples extracted from the control, nanopure water samples (Table 2 and Fig 3). Comparisons of differences in amplification efficiency between standard curves and extracted DNA among DNA targets for a positive control and four fish species were not statistically different when assessed with a one-way ANOVA, except between the Arctic Grayling and Slimy Sculpin gene targets (p < 0.0001) and Dolly Varden and Slimy Sculpin gene targets (p = 0.0038) (Fig 4).

**Fig 3:**
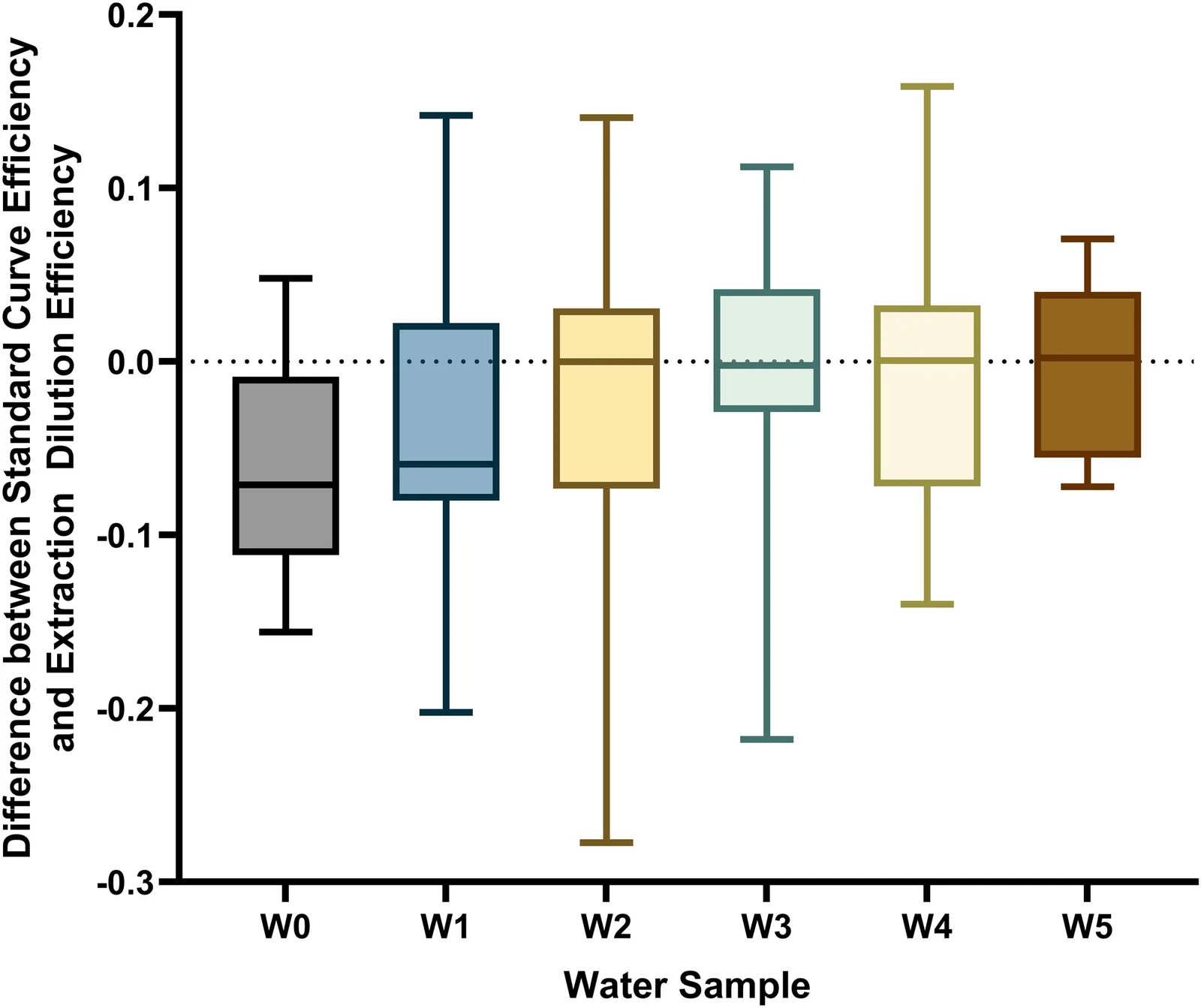
The difference between amplification efficiencies of standard curves and diluted DNA extracted from aliquots of five riverine water samples (W1-W5) affected by acid rock drainage and a purified water control (W0). Boxes are drawn from the 25^th^ percentile to the 75^th^ percentile, with the midline representing the median. Whiskers extend from the minimum observation to the maximum observation. The dashed line at y = 0 illustrates where no difference between standard curve efficiency and sample efficiency would be.

**Fig 4:**
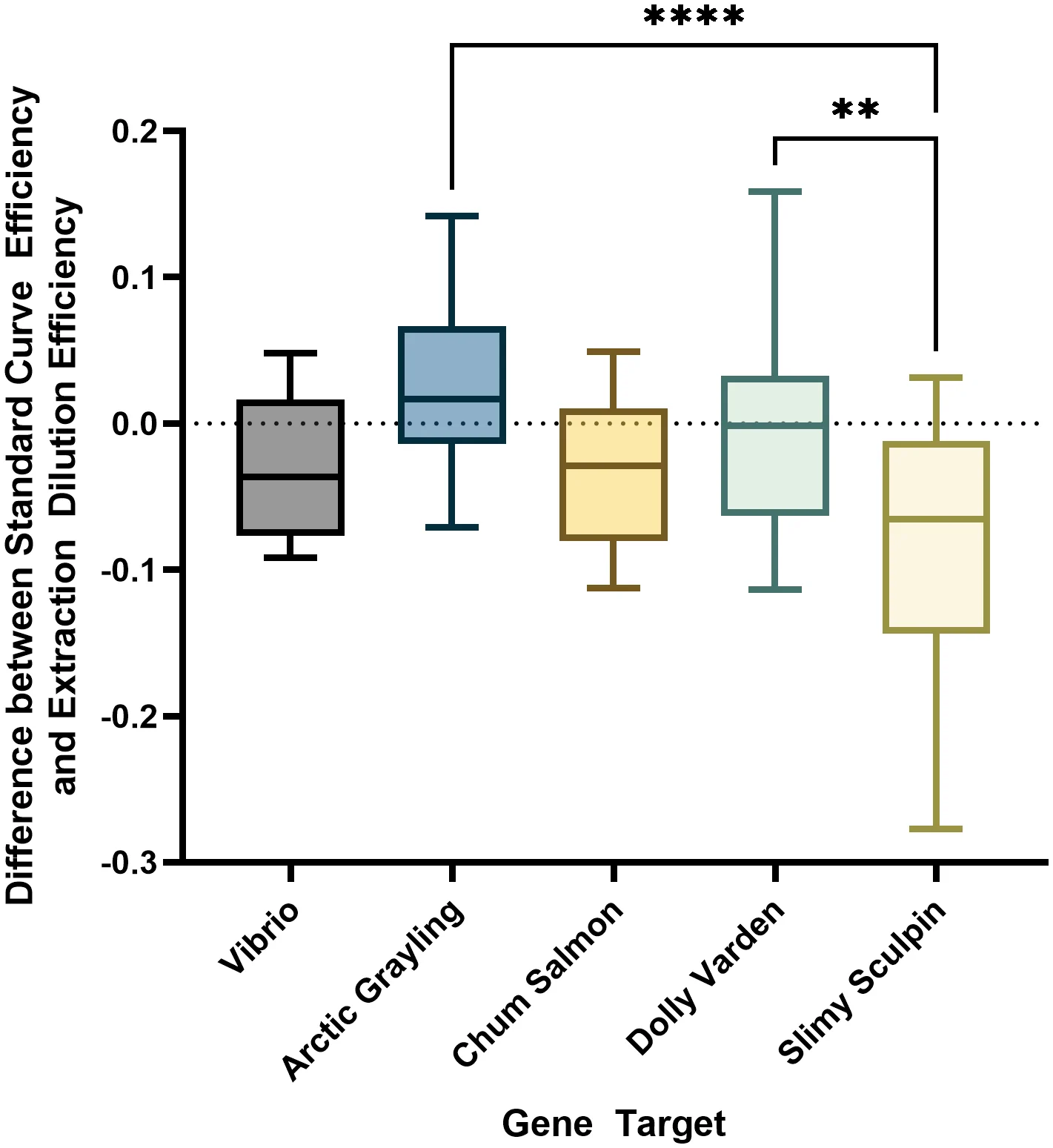
The difference between amplification efficiencies from the standard curve and DNA extracted from aliquots of five riverine water samples affected by acid mine drainage and a purified water control for qPCR amplicons of *Vibrio spp.*, Arctic Grayling (*Thymallus arcticus*), Chum Salmon (*Oncorhynchus keta*), Dolly Varden (*Salvelinus malma*), and Slimy Sculpin (*Cottus cognatus*). Boxes are drawn from the 25^th^ to the 75^th^ percentiles, with the midline representing the median. Whiskers extend from the minimum to the maximum observations. Two and four asterisks represent statistical significance at an alpha equal to 0.01 and 0.0001, respectively.

### Quantitative PCR assay efficacy using simulated mixed species assemblages

The ability of the qPCR assays to detect and proportionally quantify fish DNA from different species present in waters affected by acid rock drainage was assessed through a series of single blind tests. Of 72 species detection tests conducted using 18 water sample aliquots mixed with tissues of up to four fish species (target and off-target), there were 60 out of 60 accurate species detections, indicating a sensitivity of 100%. There was one false positive detection, or a specificity of 91.67%. The single false positive detection was for the Slimy Sculpin gene target in the laboratory control purified water treatment. The proportion of fish tissue added to water sample aliquots for each species was compared to the detected proportion of gene copies in each mixture to assess the proportional quantitative accuracy of the qPCR assays. Using a Spearman R test, the proportion of tissue added by weight and the estimated quantity of gene copies detected by qPCR were significantly correlated for Arctic Grayling (r = 0.8450; p < 0.0001), Chum Salmon (r = 0.9437; p < 0.0001), and Slimy Sculpin (r = 0.9532; p < 0.0001) (Fig 5). The proportion of Dolly Varden tissue added to a mixture was not significantly correlated with the detected concentration of Dolly Varden gene copies (r = 0.4298; p = 0.0751). The qPCR Dolly Varden assay tended to underestimate the proportion of target gene copies compared to the proportion of tissue added to a simulated mixed species assemblage (S6 Fig).

**Fig 5:**
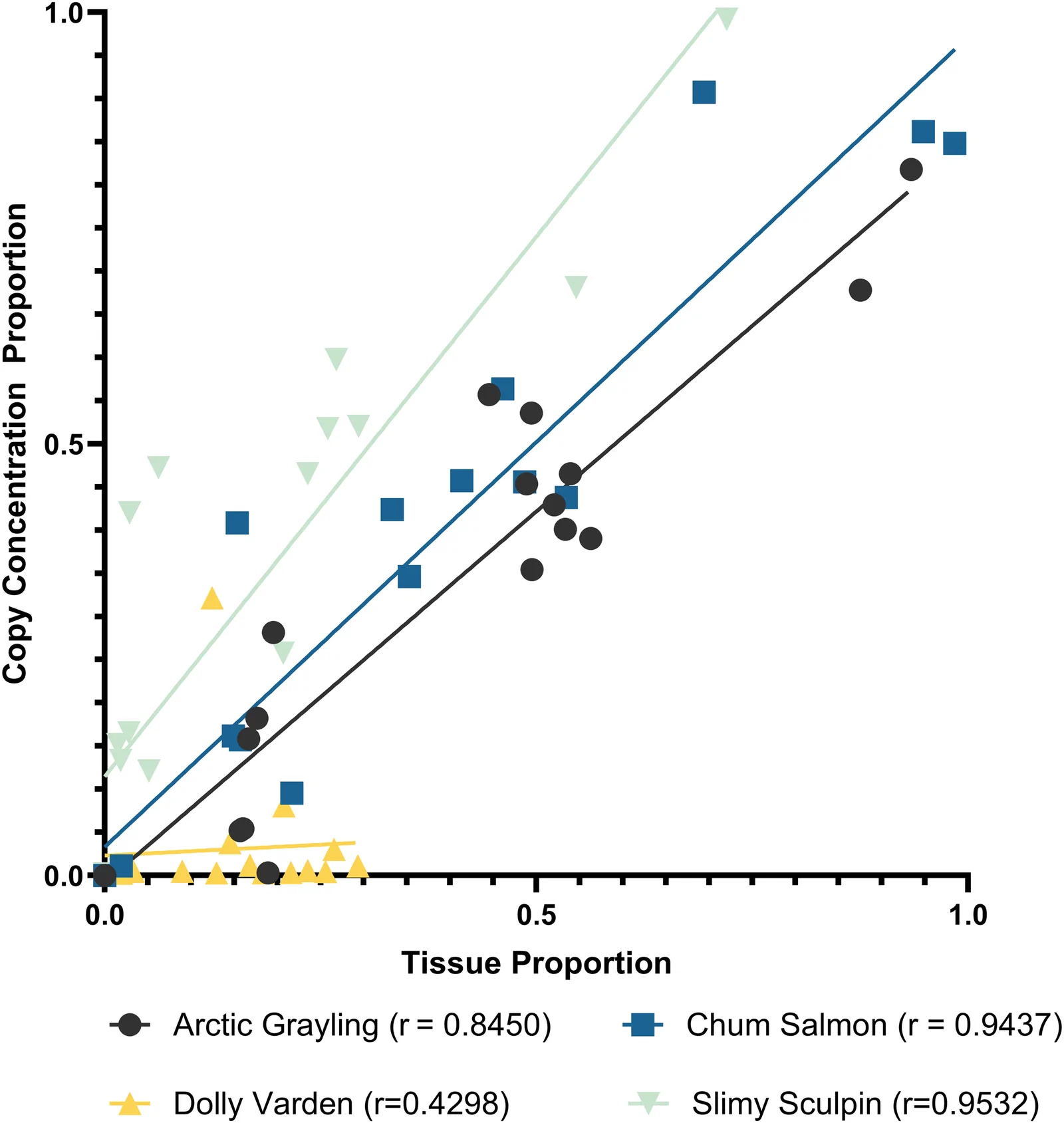
Proportion of tissue of Arctic Grayling (*Thymallus arcticus*), Chum Salmon (*Oncorhynchus keta*), Dolly Varden (*Salvelinus malma*), and Slimy Sculpin (*Cottus cognatus*) added to water mixtures and proportion of gene copy concentrations of the same species detected with quantitative PCR. The trend lines represent simple linear regression of the two proportions, with R^2^ values for each line shown in the legend.

## Discussion

Environmental DNA (eDNA) approaches are quickly being accepted as scientific tools capable of answering diverse questions regarding the presence and distribution of freshwater organisms, including economically and culturally important fish species. However, many physical (e.g., water chemistry, temperature, streamflow) and biological (e.g., organism life stage, size, movement patterns) factors may influence eDNA detection and quantification in field settings. Controlled laboratory research can, therefore, reduce uncertainty regarding efficacy and rigor of eDNA approaches prior to application in field environments where physical and biological factors are inherently difficult or impossible to control.

Our results provide evidence to support further investigation of using qPCR for field detection of eDNA for four fish species common throughout Arctic Alaska, including freshwaters that are impacted by metal mobilization from acid rock drainage [1,7]. Our qPCR approach provided consistent, accurate, sensitive, and specific detection of species-specific mitochondrial DNA fish targets highlighting the potential utility of this approach for assessing fish presence/absence. Gene copy concentrations were generally positively associated with tissue quantity added to surface water samples, but were not always significantly correlated, warranting further investigation and optimization for abundance estimation applications. By comparing tissue gene copy estimations to added tissue weights and utilizing *Vibrio* DNA spikes, we found little evidence for PCR inhibition, which is consistent with prior qPCR eDNA detections of four fish species in Canada downstream of mines [25]. We also found support for higher amplification efficiency in roughly half of the tests conducted using riverine water samples affected by acid rock drainage which is concordant with previous findings that some metal ions can improve detectability of eDNA with qPCR [18]. Some metal ions, including K+, Na+, and Mg^2^+ can improve PCR efficiency due to their role in stabilizing DNA structure [26], whereas Zn, Cu, and Fe(II) have demonstrated extensive inhibitory potential [27]. Overall, our study indicates that riverine water affected by acid rock drainage, which influences the pH, dissolved metal concentration, and particulate Fe(III) minerals [1], is likely not a constraint for applying qPCR eDNA approaches for describing fish species assemblages within Arctic Alaska. However, we acknowledge that our laboratory-based approach using muscle tissue added to autoclaved water samples is not equivalent to detecting naturally occurring eDNA in field-collected samples, such that further field validation efforts may be warranted.

Our study expands on the assays developed by Homel et al. (2020) [21] and Rodgers et al. (2018) [20] by using SYBR-based fluorescence with a master mix designed to minimize PCR inhibition and melt curves for specificity assurance. By using SYBR in the qPCR assays, amplification efficiency increased and the limits of detection were lowered in this study. SYBR green assays can be affected by non-specific amplification of primer-dimers and off-target amplicons; however, high-resolution melt temperature analysis produces specificity results comparable to results from probe-based assays [28]. Therefore, there is evidence for the utility of the SYBR-based qPCR assays used in our laboratory-controlled tests for future field-based assessments of mixed species assemblages of fishes in Arctic Alaska.

In a series of single blind tests to assess qPCR assay efficacy using simulated mixed species assemblages, we observed that the proportion of species-specific gene copies detected by qPCR was significantly correlated to the proportion of tissue added to water sample aliquots for three of the fish targets (Arctic Grayling, Chum Salmon, and Slimy Sculpin). However, experiments conducted with Dolly Varden underestimated the abundance of gene copies based upon the proportion of tissue added to the samples. No clear trend emerged regarding additional target or off-target fish tissues in the mixture or water sample when investigating the proportional differences for the Dolly Varden assay. Due to the small size of specimens used in laboratory experiments, the smallest weights of tissue added to mixtures were consistently from Dolly Varden, potentially increasing error associated with gene target quantitation. This bias in tissue preparation might be driving the proportional detection differences identified in this study. Additionally, only muscle tissue was used in this study to generate DNA targets, which likely does not represent the full spectrum or distribution of eDNA sources in most aquatic environments. Therefore, caution may be warranted if extrapolating our findings regarding the performance of qPCR assays to environmental sample conditions. Quantifying multicellular organisms with qPCR can be imprecise and unreliable due to both variance in environmental and assay conditions; however, evaluation of biomass has yielded positive results for fish abundance estimates [29–31]. Though outside the scope of the current investigation, future studies aiming to develop indices of fish abundance in rivers affected by acid rock drainage or other freshwater systems could consider digital PCR because the quantitation is absolute, more precise and accurate than qPCR, less susceptible to PCR inhibition than qPCR, and has been demonstrated to be a reliable tool for biomass estimations [16,32,33].

Our findings support that across the conditions tested (S1 Table), Arctic fish species may be reliably detected in river waters influenced by acid rock drainage using qPCR. No effect on qPCR detection occurred despite a large range of pH values (4.7 and 8.0) and elevated concentrations of metals including SO_4_^2-^, Al, Mn, Fe, and Zn commensurate with other degraded watersheds and above clearwater rivers in Arctic Alaska [1,7]. We did not systematically assess correlations between concentrations of specific metals and mineral phases and assay efficiency, which may vary between rivers beyond the conditions tested here. On average, qPCR amplification efficiencies did not change significantly in the presence of degraded water. The three observed instances of PCR inhibition occurred in the Slimy Sculpin assay, indicating this assay could be particularly susceptible to biotic and abiotic factors affecting PCR efficiency. Although we provide evidence that qPCR is a promising tool for future well-designed field-based applications to identify mixed fish species assemblages in Arctic Alaska, evaluation of qPCR methods from this study using field-collected samples could help determine the effectiveness of these approaches in natural systems. Future efforts could also be designed and pursued to better resolve how differing concentrations of mobilized metals and precipitated minerals may influence qPCR assays for specific organismal gene targets.

In summary, qPCR eDNA approaches show potential for conducting ecological assessments of mixed fish species assemblages in rivers and streams impacted by acid rock drainage from permafrost thaw in Arctic Alaska. This study represents critical initial steps toward tracking the effects of acid rock drainage on aquatic biodiversity, which can be profound, including exceeding toxicity thresholds for fish, declines in benthic macroinvertebrate density and richness, smothering periphyton biomass, and loss of habitat [1]. Quantitative PCR for four species-specific fish targets yielded sensitive, specific, and reproducible results without consistent evidence of PCR inhibition. This study provides an experimental foundation for future efforts applying qPCR eDNA tools to assess the occurrence of culturally and ecologically important fish species in aquatic Arctic environments as well as other aquatic species in watersheds affected by acid rock drainage. Furthermore, through our laboratory-based demonstration of qPCR as a way to detect fish in surface water influenced by acid rock drainage, the data summarized here contributes to growing evidence that eDNA approaches warrant consideration as appropriate methods for evaluating effects of disturbance relative to ecological change.

## Supporting information

S1 Table Study design. Water sample identification codes, geographic location of the river, collection date, and chemical parameters of water samples taken for this study including pH, temperature, dissolved oxygen (DO), specific conductance (SpC), dissolved organic carbon (DOC), dissolved inorganic carbon (DIC), chloride (Cl), sulfate (SO_4_^2-^), aluminum (Al), manganese (Mn), iron (Fe), nickel (Ni), copper (Cu), zinc (Zn), lead (Pb), cobalt (Co), magnesium (Mg), calcium (Ca), potassium (K), and sodium (Na), as well as the log Q/K of three iron(III) minerals, which is a term used to identify the saturation index of the mineral. More information can be found in O’Donnell et al. (2026)*. *O’Donnell, J.A., Koch, J.C., Carey, M.P., Poulin, B.A., Evinger, T., Frei, R.J., and Pruitt, A.N., 2026, Chemical composition of water in lakes, ponds, rivers, and creeks in northwest Alaska: U.S. Geological Survey data release, https://doi.org/10.5066/P13EV8UD.
(DOCX)

S2 Table qPCR primer and probe sequences and concentraions. qPCR primer and probe sequences and concentrations for five targets.
(DOCX)

S1 Fig Melt temperatures for qPCR amplicon product. Average melt temperatures of qPCR amplicon product between synthetic DNA templates (gBlocks) and DNA extracted from A) Arctic Grayling (Thymallus arcticus), B) Chum Salmon (Oncorhynchus keta), C) Dolly Varden (Salvelinus malma), and D) Slimy Sculpin (Cottus cognatus).
(TIF)

S2 Fig Gene copy concentrations for Arctic Grayling. Estimated gene copy concentrations from six water samples mixed with known weights of Arctic Grayling (Thymallus arcticus) tissue and analyzed with quantitative qPCR. Each dot is an individual qPCR replicate concentration estimate.
(TIF)

S3 Fig Gene copy concentrations for Chum Salmon. Estimated gene copy concentrations from six water samples mixed with known weights of Chum Salmon (Oncorhynchus keta) tissue and analyzed with quantitative qPCR. Each dot is an individual qPCR replicate concentration estimate.
(TIF)

S4 Fig Gene copy concentrations for Dolly Varden. Estimated gene copy concentrations from six water samples mixed with known weights of Dolly Varden (Salvelinus malma) tissue and analyzed with quantitative qPCR. Each dot is an individual qPCR replicate concentration estimate.
(TIF)

S5 Fig Gene copy concentrations for Slimy Sculpin. Estimated gene copy concentrations from six water samples mixed with known weights of Slimy Sculpin (Cottus cognatus) tissue and analyzed with quantitative qPCR. Each dot is an individual qPCR replicate concentration estimate.
(TIF)

S6 Fig Differences between tissue weight and detect target genes. The difference between proportion of Arctic Grayling (Thymallus arcticus), Chum Salmon (Oncorhynchus keta), Dolly Varden (Salvelinus malma), and Slimy Sculpin (Cottus cognatus) tissue by weight in water sample aliquots with simulated mixed species assemblages and the proportion of detected target gene copies using qPCR. Individual observations are shown with dots. Boxes are drawn to the median and error bars illustrate the standard deviation.
(TIF)
