# Supplementary material for: Laboratory-based assessment of quantitative PCR to describe Arctic fish assemblages in surface water affected by acid rock drainage
Source: PLoS One. 2026 Nov 10;21(11):e0362179. doi: 10.1371/journal.pone.0362179 (PMC13750573; doi:10.1371/journal.pone.0362179)
Supplement: Supplementary file 1 [file pone.0362179.s001.docx]

| **Sample ID** | **W1** | **W2** | **W3** | **W4** | **W5** | **W0** |
| --- | --- | --- | --- | --- | --- | --- |
| **Watershed** | **Igning River** | **Igning River** | **Igning River** | **Igning River** | **Kugururok River** | **Nanopure Control** |
| **Latitude** | **67.613124** | **67.537029** | **67.548046** | **67.54142** | **68.342942** | **--** |
| **Longitude** | **-155.920130** | **-155.933457** | **-155.918680** | **-155.9338** | **-161.352461** | **--** |
| **SiteID** | **Mainstem** | **Mainstem** | **Mainstem** | **Unnamed tributary** | **Unnamed tributary** | **--** |
| **Date** | **8/26/2024** | **8/26/2024** | **8/26/2024** | **8/26/2024** | **6/3/2024** | **--** |
| **Sampl Time (h:mm)** | **15:30** | **14:40** | **15:20** | **14:30** | **16:20** | **--** |
| pH | 8.03 | 7.66 | 7.78 | 6.77 | 4.78 | -- |
| Temperature (°C) | 6.2 | 5.7 | 5.8 | 5.8 | 2.9 | -- |
| DO (mg/L) | 11.41 | 11.23 | 11.14 | 11.19 | 13.37 | -- |
| SpC (μS/cm) | 1505 | 1219 | 1514 | 3469 | 1397 | -- |
| DOC (mg/L) | 0.59 | 1.52 | 0.91 | 0.69 | 1.26 | -- |
| DIC (mg/L) | 41.71 | 40.35 | 41.13 | 12.45 | 0.68 | -- |
| Cl^-^ (mg/L) | 16.1 | 1.26 | 2.03 | 4.17 | 4.14 | -- |
| SO_4_^2-^ (mg/L) | 744 | 594 | 734 | 2404 | 916 | -- |
| Unfilt_Al (μg/L) | 1390.8 | 2352.7 | 1845.9 | 17132.6 | 13595.3 | -- |
| Unfilt_Mn (μg/L) | 499.7 | 625.5 | 803.5 | 5692.9 | 27194.0 | -- |
| Unfilt_Fe (μg/L) | 4507.9 | 3638.2 | 7441.6 | 86636.9 | 28859.8 | -- |
| Unfilt_Ni (μg/L) | 123.8 | 163.8 | 192.2 | 1132.7 | 1183.4 | -- |
| Unfilt_Cu (μg/L) | 29.0 | 65.9 | 55.9 | 330.6 | 86.8 | -- |
| Unfilt_Zn (μg/L) | 553.5 | 1028.6 | 961.8 | 3364.5 | 3184.1 | -- |
| Unfilt_Pb (μg/L) | 0.81 | 1.11 | 0.27 | 1.92 | 0.47 | -- |
| Unfilt_Co (μg/L) | 14.6 | 19.8 | 23.6 | 148 | 202 | -- |
| Filt_Al (μg/L) | 144 | 41.9 | 69.9 | 40.2 | 12880 | -- |
| Filt_Mn (μg/L) | 542 | 19.2 | 22.6 | 6423 | 30307 | -- |
| Filt_Fe (μg/L) | 69.8 | 16.8 | 34.2 | 257 | 22270 | -- |
| Filt_Ni (μg/L) | 95.9 | 4.61 | 4.70 | 25.5 | 1291 | -- |
| Filt_Cu (μg/L) | 1.16 | 0.073 | 0.048 | 0.175 | 71.1 | -- |
| Filt_Zn (μg/L) | 172 | 13.8 | 7.41 | 46.0 | 3483 | -- |
| Filt_Pb (μg/L) | 0.052 | 0.010 | 0.007 | 0.034 | 0.150 | -- |
| Filt_Co (μg/L) | 11.4 | 0.570 | 0.609 | 3.37 | 199 | -- |
| Filt_Mg (mg/L) | 125.00 | 92 | 127 | 416 | 111.8 | -- |
| Filt_Ca (mg/L) | 150.00 | 127 | 151 | 318 | 100.1 | -- |
| Filt_K (mg/L) | 0.00 | 0.00 | 0.00 | 0.00 | 0.00 | -- |
| Filt_Na (mg/L) | 0.28 | 0.16 | 0.14 | 0.72 | 7.6 | -- |
| Sat_Index_Fe2O3 (logQ/K) | 13.16 | 11.9 | 12.5 | 13.7 | 13.9 | -- |
| Sat_Index_FeO(OH) (logQ/K) | 6.13 | 5.5 | 5.81 | 6.4 | 6.51 | -- |
| Sat_Index_Fe(OH)3 (logQ/K) | 1.52 | 0.88 | 1.2 | 1.77 | 1.87 | -- |
