# Supplementary material for: Laboratory-based assessment of quantitative PCR to describe Arctic fish assemblages in surface water affected by acid rock drainage
Source: PLoS One. 2026 Nov 10;21(11):e0362179. doi: 10.1371/journal.pone.0362179 (PMC13750573; doi:10.1371/journal.pone.0362179)
Supplement: Supplementary file 2 [file pone.0362179.s002.docx]

| Species | Common name | Forward | Reverse | Probe | [C nM](F/R/P) |
| --- | --- | --- | --- | --- | --- |
| *Thymallus arcticus* | Arctic grayling | TGTGGGCTGTTCTGATTACCG | TGCTGGGTCAAAGAAAGTGGTATTA | CTTGCAGCAGGTATC | 600/600/250 |
| *Oncorhynchus keta* | Chum salmon | CCGCTTTTTGTCTGAGCTGTACT | AATTTCGATCTGTGAGCAACATAGTAA | CTGTACTTCTACTATTATCACTCC | 300/900/250 |
| *Salvelinus malma* | Dolly varden | CCGCCACAGTACTTCACCTTCTA | AGGCCAAGCAATATAGCTACGAAA | CCGACAAAATCTCATTCC | 100/600/250 |
| *cottus cognatus* | Slimy sculpin | GGAGGCGTCCTAGCCCTC | GAGTCCAAAATAGGAATTGGGTCAC | CATCCATCCTGGTGCTCAT | 300/900/250 |
| *Vibrio spp.* | NA | CTACTTGGAGGTTGTGGCCT | GCTGGCAAACAAGGATAAG | NA | (500/500/NA) |
